# Bacterial Etiology of Urinary Tract Infection and Antibiogram Profile in Children Attending Debre Tabor Comprehensive Specialized Hospital, Northwest Ethiopia

**DOI:** 10.1155/2023/1035113

**Published:** 2023-08-01

**Authors:** Teklehaimanot Kiros, Melaku Zeleke, Tahir Eyayu, Lemma Workineh, Shewaneh Damtie, Tesfaye Andualem, Tegenaw Tiruneh, Ayenew Assefa, Sisay Getu, Tazeb Molla, Tsehaynesh Gebreyesus

**Affiliations:** ^1^Department of Medical Laboratory Sciences, College of Health Sciences and School of Medicine, Debre Tabor University, Debre Tabor, Ethiopia; ^2^Debre Tabor College of Health Sciences, Debre Tabor, Ethiopia; ^3^Amhara Public Health Institute, Bahrdar, Ethiopia

## Abstract

**Background:**

Bacterial urinary tract infections are important public health problems in children. This study was conducted to identify the bacterial agents of urinary tract infections and antibiogram patterns in children.

**Methods:**

A hospital-based cross-sectional study including 220 children was carried out between November 15, 2021, and March 10, 2022. Simple random sampling was used to enroll participants. The sociodemographic and clinically pertinent information was gathered using a semi-structured questionnaire. Every participant in the study who was ≤15 years old gave clean-catch midstream urine. Urine samples were inoculated onto a cystine lactose electrolyte-deficient agar using a calibrated inoculating loop with a 0.001 ml capacity and then incubated aerobically for 24 hours at 37°C. Subculturing for significant bacteriuria was done on MacConkey and blood agar. Gram staining, biochemical assays, and colony characteristics were used for bacterial identification. The disc diffusion method developed by Kirby and Bauer was used for antimicrobial susceptibility testing. SPSS software version 25 was used for data entry and analysis. To find the risk factors, bivariate and multivariate logistic regression analyses were performed. An association was deemed statistically significant if the *p* value at the 95 percent confidence interval was less than 0.05.

**Results:**

In this study, the majority (50.5%) of the study participants were males. The mean age of the study participants was 6 ± 0.91 years. It was found that 31.8% of children had urinary tract infections. The most prevalent urinary pathogens among the isolates were *E. coli* (27.1%) and *S. aureus* (18.6%). Approximately 56% of the participants were infected with multidrug-resistant pathogens. Additionally, compared to children who have never had a urinary tract infection, children with a history of infection had 1.04 (95 percent confidence interval (CI): 0.39, 2.75) times higher risk of infection.

**Conclusion:**

This study has shown an alarming increase in the prevalence of pediatric urinary tract infections which warrants further investigation into multidrug-resistant bacterial infection.

## 1. Introduction

Urinary tract infections (UTIs) caused by bacteria are among the most prevalent nosocomial illnesses. They are responsible for rising mortality, morbidity, and medical costs over the world [1, 2]. Infection includes the kidneys, bladder, ureters, and urethra. The bladder and kidneys become infected when uropathogenic bacteria from the skin or rectum pass via the urethra. It is a common infection in pediatric patients and can cause various symptoms such as frequent urination and pain or burning during urination [3, 4]. The infection site, complication status, and environment where the infection was obtained are considered for classification. According to epidemiological characteristics, it can also be classified as a community-acquired UTI or a healthcare-associated UTI [1, 3, 5].

Bacterial urinary infections are responsible for more than 150 million cases worldwide [6]. According to estimates, the burden of UTI is significant in terms of healthcare expenses and lost productivity. The annual overall communal costs are substantial and amount to about US $3.5 billion [1]. Data from developing countries have shown that nearly 10% of children with febrile illnesses have UTI but can be extended to 8–35% among malnourished children [7]. Two cross-sectional studies done in 2017 and 2019 from Nepal [8, 9] have shown a prevalence of 15.88% and 57%, respectively. Similarly, one study in India also reported 48% prevalence [10]. According to the global outlook for 2015, it is one of the most prevalent causes of febrile illness in children, with a global prevalence of 2–20% [11, 12]. Globally, about 8% and 2% of girls and boys, respectively, have experienced at least one episode of UTI by the age of 7 years [11, 13]. This can be associated with high mortality, morbidity, and long-term complications such as renal scarring, hypertension, and chronic renal failure [12, 14]. Moreover, pediatric UTI is becoming underdiagnosed, primarily because there are no clear indications or symptoms. According to estimates, pediatric patients experience a 50% miss rate for infections (including silent and symptomatic cases) [3, 15].

Both Gram-positive and Gram-negative bacteria are involved in the infection process. The most common causative agents for both uncomplicated and complicated urinary infection include *Escherichia coli*, *Klebsiella pneumoniae*, *Staphylococcus saprophyticus*, *Enterococcus faecalis*, *Proteus mirabilis*, *Pseudomonas aeruginosa*, and *Staphylococcus aureus* [5]. A study in Iran [16] has shown that uropathogenic *E. coli* was the most common isolate (51.5%) followed by *Klebsiella* spp. (16.8%) and *Enterococcus* spp. (9.9%). Another study conducted in Nepal [12] revealed that *E. coli* was the most predominant etiologic agent (53%) followed by *E. faecalis* (22%), *K. pneumoniae* (7%), and *S. aureus* (7%). Similarly, a study conducted in Greece [17] reported that 170 (76.9%) *E. coli* followed by 17 (7.7%) *Proteus* spp., 15 (6.8%) *Klebsiella* spp., 9 (4.1%) *P. aeruginosa*, 4 (1.8%) *E. faecalis*, 2 (0.9%) *Enterobacter* spp., and 2 (0.9%) *Morganella morganii* were the pathogens most frequently found.

The most serious issue with regard to public health is antimicrobial resistance (AMR). It has the potential to reduce the effectiveness of antibacterial medicines. Infections especially those brought on by drug-resistant microbes also raise morbidity, death, and healthcare expenditures [18, 19]. Children receive a disproportionately high amount of antibiotics compared to adults, which makes them vulnerable to the rising AMR issue [20]. Accurate diagnosis and prompt prescription of antimicrobial drugs are of the utmost importance and a top priority in the prevention and control of AMR transmission dynamics in healthcare settings [11, 12, 21].

Controlling infections necessitates research into the risk factors for UTI in children. Socioeconomic and clinical or treatment-related factors are among the others. Consequently, addressing these issues enhances the management and treatment of UTI in children [1, 5, 7]. There has not been much research done in Ethiopia on the bacterial uropathogens and the patterns of antibiotic resistance in children. Only fewer published studies have shown a prevalence of 27.5% [22], 15.9% [23], 26.45% [24], and 16.7% [7], respectively. To our knowledge, there is no published information on the bacterial profile, antimicrobial susceptibility patterns, and the risk factors connected in the study area. AMR is a very dynamic phenomenon and a quickly growing global public health concern. Generating local epidemiological and clinical data could assist to precisely diagnose and select the best treatment options. Moreover, the results of this study should assist public health experts in making an informed decision over empiric therapy.

## 2. Methods and Materials

### 2.1. Study Area, Design, and Period

A hospital-based cross-sectional study was conducted from November 15/2021 to March 10/2022 among pediatric patients attending Debre Tabor Comprehensive Specialized Hospital (DTCSH), Northwest Ethiopia. Debre Tabor is located in the southwest of the Gondar Zone in the Amhara Region. It is about 666 kilometers away from Addis Ababa, the capital city of Ethiopia. The town has a latitude and longitude of 11°51′N 38°1′E, respectively, with an elevation of 2706 meters above sea level. DTCSH is currently providing a comprehensive service in collaboration with Debre Tabor University, College of Health Sciences, and School of Medicine. It is serving more than one million of the population coming to seek health services with an annual average client flow of 650,531. The hospital has the capacity to hold more than 400 beds, has more than 250 healthcare providers, and has more than 100 administrative and technical staff. It is providing a service for a large number of people coming from the surrounding zones for both outpatient and inpatient services (emergency, medical, surgical, obstetric/gynecologic, neonatal intensive care, pediatric, and orthopaedic). Based on the information from the hospital's health management and information system, the hospital is currently providing pediatric services for more than 250 children daily.

### 2.2. Source Population

The source population included all children who were attending DTCSH during the study period.

### 2.3. Study Population

All pediatric patients ≤15 years old who were clinically suspected of having a UTI during the specified study period comprised the study population.

### 2.4. Eligibility Criteria

#### 2.4.1. Inclusion Criteria

All pediatric patients who were clinically suspected of UTI, having age ≤15 years old, and those willing to participate in the study based on their family consent were included for bacteriological investigation. Moreover, children with positive urinalysis, especially for nitrite test, leukocyte esterase, and protein, were included. Furthermore, children with indications of pyuria (presence of ≥5 WBCs per high-power field) were included in the study.

#### 2.4.2. Exclusion Criteria

Pediatric patients who have recently received antimicrobial treatment, are presently taking antibiotics, and whose parents/guardians refused to give their agreement for their kid to participate in the study were excluded from the study. Additionally, children who were critically ill and mentally incapacitated, absent during the time of data collection, and those whose medical records were incomplete were excluded.

#### 2.4.3. Sample Size Determination and Sampling Technique

The required sample size for this study was determined based on the equation used for the estimation of a single population proportion formula = [(Z*α*/2)^2^*P* (1 − *P*)/*d*^2^)] by taking the prevalence of 16.7% [7], using a 95% confidence interval (95% CI; Z*α*/2 = 1.96) and the margin of error (*d* = 0.05). By substituting the above values, the sample size was approximately 214. By adding a 10% non-response rate (214 *∗* 0.1 = 21), the total sample size required for the study was 235. Regarding the sampling method, the study participants were chosen using a simple random sampling technique. Approximately, 50 kids on average visited each day to receive care, and a third of them simultaneously provided urine for urinalysis and urine culture.

### 2.5. Study Variables

#### 2.5.1. Dependent Variables

- Prevalence and antimicrobial susceptibility patterns.

#### 2.5.2. Independent Variables

- Age, sex, residence, patient setting, duration of hospitalization, history of catheterization, previous history of UTI, previous exposure to antibiotics, presence of chronic disease, and presence of malnutrition.

#### 2.5.3. Operational Definition

- *Sensitive (S)*: bacterial isolates are inhibited by the usually achievable concentration of antimicrobial agents [25].
- *Intermediate (I)*: bacterial isolates with an antimicrobial agent of minimum inhibitory concentration that approach usually attainable blood and tissue levels and for which response rates may be lower than those for susceptible isolates [25].
- *Resistant (R)*: bacterial isolates uninhabited by the usually achievable concentration of the agent with normal dosage [25].
- *Multidrug resistance (MDR)*: when a bacterium is simultaneously non-susceptible to three or more drugs belonging to different classes of antibiotics [26].

### 2.6. Data Collection Methods

#### 2.6.1. Sociodemographic and Clinical Data Collection

Prior to the collection of sociodemographic and clinically pertinent data, all families, care givers, and guardians of children received verbal and written participant information. Then, sociodemographic data and other pertinent clinical features were gathered using a semi-structured and pretested questionnaire. The questionnaire was initially developed in English, translated into Amharic, and then returned to English to confirm its accuracy, comprehensiveness, simplicity, coherence, and clarity. Additionally, 10% of the questions were pretested in Addis Zemen Primary Hospital prior to the study's launch. Then, taking into account the test results from the respondents, minor tweaks were made.

### 2.7. Laboratory Investigation

#### 2.7.1. Specimen Collection, Handling, Processing, and Transport

15 patients were not permitted to participate because they refused to give their consent. Prior to sample collection, the families and guardians were given the necessary instructions on how to collect a clean-catch midstream urine specimen for urinalysis and urine culture. Participants in the study and their families were also urged to supply urine samples before beginning antibacterial medication because even one round of antibiotics can have an impact on a urine culture. Since there was a high chance of contamination, urine samples collected using a collecting bag or pad were disregarded [27]. Then, 5–10 ml of urine samples was taken from each research subject using sterile, screw-capped, wide-mouth containers and labeled with the patient's medical identification number [28]. The urine sample was subsequently subjected to bacteriological testing using standard operating procedures [7, 23, 29].

#### 2.7.2. Urine Culture

Clean-voided midstream urine was employed in this investigation to identify the urinary isolates. In short, urine samples were inoculated into cystine lactose electrolyte-deficient (CLED, Oxoid, Basingstoke, Hampshire, England) agar using a calibrated sterile plastic loop with a capacity of 0.001 ml and incubated in the aerobic atmosphere at 37°C for 24 hours. Colonies were then counted to see if there was any major bacteriuria present. In actual use, 1 *μ*l loop (0.01 ml capacity) was utilized to detect colony counts between 100 and 1,000 CFU/ml, whereas a 10 *μ*l loop (0.001 ml) was employed to detect colony counts larger than 1,000 CFU/ml.

In positive cultures for a 1 *μ*l loop, one colony equals 1,000 CFU/ml, and for a 10 *μ*l loop, one colony equals 100 CFU/ml. Based on these scenarios, the maximum readable using the 1 *μ*l loop was 10^5^ CFU/ml and the maximum readable on the 10 *μ*l (0.01 ml) loop was 10^4^ CFU/ml [30]. Hence, colony count yielding bacterial growth of ≥10^5^ CFU/ml of urine was considered significant bacteriuria. Similarly, all positive urine cultures with significant bacteriuria were then subcultured to MacConkey agar (Oxoid, Basingstoke, Hampshire, England) and 5% sheep blood agar (Oxoid, Basingstoke, Hampshire, England) [7, 22, 23]. Isolate identification to species level was done by their colony characteristics (size, shape, turbidity, and hemolysis), Gram staining, and pattern of biochemical profiles. The biochemical test was conducted to assess indole production, H2S production, lactose fermentation in TSI agar, citrate utilization, motility test, urease test, bile solubility, and lysine utilization in lysine decarboxylase( LDC) agar [28]. An oxidase test was also performed for other Gram-negative rods. On the contrary, the Gram-positive bacteria were identified using catalase and coagulase tests.

#### 2.7.3. Antibiotic Susceptibility Testing (AST)

According to the Clinical and Laboratory Standards Institute (CLSI), the Kirby–Bauer disc diffusion technique was used for the antimicrobial susceptibility testing [25]. A loop full of three to five isolated colonies with identical colony morphology was collected from a pure culture, transferred to a tube holding four to five milliliters of normal saline, and gently mixed until it produced a homogenous suspension. The size of the inoculum was then standardized by adjusting the suspension's turbidity to a 0.5 McFarland standard.

A sterile cotton swab was then dipped into the suspension, and the excess was removed by gentle rotation of the swab against the surface of the tube. The swab was then used to distribute the bacteria evenly over the entire surface of Mueller–Hinton agar (MHA, Oxoid, Basingstoke, Hampshire, England). The inoculated plates are left at room temperature to dry for 3–5 minutes. Then, with the aid of sterile forceps, antibiotic discs were placed on the surface of MHA.

The following antibiotic discs obtained from Oxoid (Mast Diagnostics, UK) were used against Gram-negative bacteria isolates: amoxicillin-clavulanate acid (AMC = 20/10 *μ*g), ampicillin (AMP = 10 *μ*g), ceftriaxone (CTR = 30 *μ*g), chloramphenicol (CHL = 30 *μ*g), ciprofloxacin (CIP = 5 *μ*g), gentamicin (CN = 10 *μ*g), meropenem (MER = 10 *μ*g), nitrofurantoin (NIT = 300 *μ*g), ceftazidime (CAZ = 30 *μ*g), tetracycline (TET = 30 *μ*g), and trimethoprim-sulfamethoxazole (SXT = 1.25/23.75 *μ*g). Similarly, for Gram-positive bacteria isolates, chloramphenicol (CHL = 30 *μ*g), ciprofloxacin (CIP = 5 *μ*g), gentamicin (CN = 10 *μ*g), nitrofurantoin (NIT = 300 *μ*g), tetracycline (TET = 30 *μ*g), trimethoprim-sulfamethoxazole (SXT = 1.25/23.75 *μ*g), penicillin (PEN = 10 units), clindamycin (CLN = 2 *μ*g), ampicillin (AMP = 10 *μ*g), and erythromycin (ERY = 15 *μ*g) were used for drug sensitivity testing. The plates were inverted and incubated in the aerobic atmosphere at 37°C for 24 hours. Diameters of the zone of inhibition around the discs were measured by a ruler, and the isolates were classified as sensitive, intermediate, and resistant as per the CLSI guideline [25].

### 2.8. Data Quality Assurance

Measures for quality control (QC) were used throughout the entire laboratory workup. The use of all materials, tools, reagents, and processes was properly monitored. In addition, the sterility of culture media was checked by incubating 5% of the batch at 37°C overnight and evaluated for possible contamination. Similarly, the standard reference strains such as *S. aureus* (ATCC25923), *P. aeruginosa* (ATCC-27853), *E. coli* (ATCC-25922), *E. faecalis* (ATCC 29212), and *S. pneumoniae* (ATCC 49619) were tested weekly for biochemical tests and agar plates including MHA with antimicrobial discs to ensure testing performance or the potency of antimicrobial discs according to the American Type Culture Collection (ATCC). Moreover, result documentation and interpretation were double-checked to avoid possible transcriptional errors.

### 2.9. Data Processing and Statistical Analysis

Using SPSS version 25 software, data were entered, verified, and analyzed. Texts and tables were used to present the study's findings. Both bivariate and multivariate logistic regression analyses were carried out to address the risk factors connected to pediatric UTI, and variables with the *p* value ≤0.2 in the bivariate logistic regression analysis were imported to the multivariate logistic regression analysis. The link between possible risk variables and pediatric UTI was estimated using the adjusted odds ratio at 95 percent confidence interval (CI). Finally, a statistically significant association was defined as one with a *p* value of <0.05 at the 95% CI.

### 2.10. Ethical Clearance

The Research and Ethical Review Committee at Debre Tabor University gave its approval to this study (letter of reference: chs/1109/2021). To ensure participant privacy, all data collection process and urine culture findings were kept confidential.

## 3. Results

### 3.1. Sociodemographic Characteristics

In this study, a total of 220 participants were included. The majority of the study participants (50.5%) were males. The age category of the study participants indicated that 88 (40%) followed by 87 (39.5%) were embodied under the 5–10 and 5 years group, respectively. The mean age of the study participants was 6 years ± 0.91 standard deviations (std). Among the total, 142 (64.5%) were urban residents (Table 1).

### 3.2. Clinical Characteristics

According to the clinical characteristics of the study's participants, 154 (70%) were outpatient attendants. About 80 (36.4%) of the pediatric patients had experienced a past history of UTI. Among all study participants, 62 (28.1%) and 71 (32.3%) had experienced lengthy hospital stays and intensive care unit (ICU) stays, respectively (Table 2). Among the total of 220 urine specimens, the prevalence of pediatric UTI was 31.8% (95% CI: 29.72, 56.15).

### 3.3. Bacterial Etiologic Agents

A total of 70 bacterial isolates were reported in this study. Gram-positive and negative bacteria, respectively, account for 40% and 60% of infections (Table 3). *S. aureus* (46.4%) was the most prevalent isolate among the Gram-positive bacteria. Similarly, *E. coli* contributes 19 (27.1%) of the total isolates.

### 3.4. Antibiogram Patterns

The various classes of antibacterial drugs were evaluated against the urinary pathogens. Ten antibiotics, including penicillin, cephalosporins, carbapenems, and aminoglycosides, were tested against the Gram-positive isolates. Likewise, eleven antimicrobial drugs were evaluated against the Gram-negative bacterial isolates. Only 7 (77.8%) of the CoNS in our investigation were susceptible to clindamycin, while all *S. aureus* isolates were 100% susceptible. Penicillin and chloramphenicol were totally resistant to all six isolates of *Enterococcus* spp. (Table 4). In the Gram-positive isolates, 20 (71.4%) and 18 (64.3%) had a sensitivity to clindamycin and ciprofloxacin, respectively. On the other hand, 25 (89.3%) and 26 (92.9%) of those isolates, respectively, have shown resistance to nitrofurantoin and penicillin. According to the antimicrobial susceptibility patterns for the Gram-negative isolates, meropenem was 100% effective against every isolate of *E. coli*, *Acinetobacter* spp., and *Citrobacter* spp. Moreover, *K. pneumoniae* has demonstrated total resistance to gentamicin, ciprofloxacin, trimethoprim-sulfamethoxazole, and nitrofurantoin (Table 5).

### 3.5. Multidrug Resistance

In this study, the multidrug resistance (MDR) pattern was done based on the previously published international guideline [26]. Based on this, thirty-nine (*n* = 39) of the isolates were resistant to three and more antimicrobial agents belonging to different classes. Therefore, the overall prevalence of MDR was 55.7% (95% CI: 32.26, 76.09). The MDR patterns of the urinary isolates are also summarized in Table 6.

### 3.6. Associated Risk Factor Analysis

To determine the independent factors linked to the probability of developing pediatric UTI, the risk factor analysis was carried out (Table 7). Bivariate logistic regression analysis was analyzed for all variables. The multivariate logistic regression model was then used to identify possible risk factors. According to the analysis of this study, children with a prior history of UTI had 1.04 (95% confidence interval: 0.39–2.75) times higher risk of developing UTI than children without such a history. The probability of developing a UTI was also 2.18 (0.14–5.13) times higher in underweight children than in well-nourished children. As a result, there is a statistically significant correlation between the two variables and the likelihood of UTI.

## 4. Discussion

The bacterial origin of UTI is responsible for causing infections among children. Early diagnosis of urinary pathogens and prompt treatment can significantly reduce the complications affecting the lives of many children [31]. Otherwise, a late or undiagnosed UTI can increase negative impacts like morbidity, mortality, and medical costs [17].

In this study, the overall prevalence of pediatric UTI was 31.8%. The current finding is significantly higher than the results of earlier research, such as those from Addis Ababa 15.8% [23], Bahrdar 16.7% [7], Iran 16.2% [32], two studies from Nepal (16% and 19.68% [11, 12], respectively) and Nigeria 11.96% [33]. Likewise, considerably lower outcomes were reported from Iran (3.6% and 7.87% [34, 35], respectively) India 4% [36], America 7% [37], and Nigeria 3% [38]. However, the present finding is consistent with the research conducted in Gondar 26.45% [24], Iran 50.5% [16], and Egypt 41.3% [39]; however, much lower than the study done in Turkey 100% [21]. The discrepancies in the magnitude of UTI may be attributed due to the difference in study population [16, 40], study sample size [32, 35, 36], study area, setting [12, 17, 41], and study design and period [11, 36, 42].

In this study, both the Gram-positive isolates and Gram-negative isolates were detected. According to the distribution of the isolates, the most common urinary pathogens among children were E. coli 19 (27.1%), S. aureus 13 (18.6%), and K. pneumoniae 9(12.9%). Among the Gram-negative isolates, *E. coli* accounts for 45.2%, whereas *S. aureus* contributes 46.4%. Similar to the present finding, a study in Addis Ababa [23] reported that *E. coli* (49.5%) was the most predominant isolate followed by *Klebsiella* spp. (27.9%), *S. aureus* (8.2%), and *Enterococcus* spp. (11.5%). Another study from Gondar [24] has shown that *E. coli* (54.88%) was the most frequently detected urinary pathogen followed by *S. aureus* (9.75%), *P. aeruginosa* (4.88%), and *Enterococcus* species (3.66%). Moreover, another study [7] has revealed that *E. coli* (63.6%) was the most dominant isolate among the Gram-negative isolates while the CoNS (among others, *S. saprophyticus*, 33.3%) was more prevalent among Gram-positive bacterial uropathogens. Furthermore, the present study has shown similar findings to many other studies including [11, 12, 16, 17, 21, 35].

Regarding the AST patterns, all isolates of S. *aureus* were susceptible to clindamycin (100%) and all isolates of *Enterococcus* spp. were completely resistant to penicillin and chloramphenicol. Additionally, the Gram-negative isolates have shown that all isolates of *E. coli*, *Acinetobacter* spp., and *Citrobacter* spp. were completely susceptible to meropenem (100%) while the majority of isolates of *K. pneumoniae* showed resistance to nitrofurantoin, trimethoprim-sulfamethoxazole, ciprofloxacin, and gentamicin. Aligning to the present study, a study in Brazil [41] reported that Gram-negative isolates, particularly *E. coli*, indicated a high resistance against trimethoprim-sulfamethoxazole (51%). In agreement with the present study, another study from Greece [17] has indicated that the majority of Gram-negative isolates were susceptible to ampicillin and amoxicillin-clavulanate acid. Contrary to the current finding, a study from Iran [16] reported that both Gram-negative and positive bacteria isolates have shown the highest resistance against amoxicillin (83.8%) and clindamycin (100%), respectively. In another study [35] in contrast to present findings, it was revealed that ciprofloxacin is 100% active against Gram-negative isolates including *Klebsiella* spp. and *P. aeruginosa* isolates followed by amikacin, nalidixic acid, and gentamicin. The variation might be due to the market availability of antibiotic discs [35] provided that nalidixic acid was not tested in the present study, the production of extended-spectrum beta-lactamase or carbapenemases [11, 16], and the presence of highly virulence factors that enable uropathogenic isolates to withstand resistance for cephalosporins and carbapenems [40]. Moreover, the difference in the study area, the level of infection prevention and control [43], the socioeconomic condition of the study population, and the influence of the prevalence of drug resistance pathogens across the globe will contribute to the disparity [44].

In this study, the overall prevalence of MDR was 55.7%. Nearly concordant results with the present MDR rate were found like 58.54% [24], 66% [7], 64.9% [11], and 50% [35]. However, data that were slightly different from the current findings, such as 73.7% [23] and 32% [12], were also reported. The difference in the prevalence of MDR can be explained by the difference in definition of MDR given that the present study adheres to the previously published international guideline, irrational consumption of antimicrobials in human and animal settings, the pathogen's virulence factors [19], lack of periodic surveillance for the emerging MDR in clinical settings [45], community, societal, and economic standing [17], and excessive empirical therapy especially cephalosporins and carbapenem regimens [11].

Another important issue of this study is the risk factor analysis. The multivariate logistic regression model has revealed that children having a previous history of UTI were 1.04 (95% CI: 0.39–2.75) times at more risk to develop UTI compared with those who have no infections. In the meantime, malnourished children were 2.18 (95% CI: 0.14–5.13) times at greater risk for the acquisition of UTI compared with normally nourished. Similar to our findings, one study [22] reported that under-nourished children were 5.41 (95% CI: 2.64–11.07) times at higher risk of contracting UTI compared with those who were normally nourished. This may be due to the fact that severe malnutrition can make children susceptible to different infections including UTIs by diminishing their immunity. Also, the children with a past history of UTI were 1.04 times at more risk to contract UTI compared with those who have no UTI. Similar results were found in [7, 24].

### 4.1. Strengths and Limitations

This study focused on the profile of bacterial uropathogens, their antibacterial patterns, and the prevalence of UTIs in children. Instead of depending on empirical treatment, it will make it easier to provide appropriate treatment through the systematic selection of antibiotics based on knowledge of the local epidemiological and clinical data. This study has some drawbacks. The multidrug resistance caused by urinary pathogens that produce extended-spectrum beta-lactamases and carbapenemases was not covered in this investigation.

## 5. Conclusion

In general, children had UTIs more commonly from Gram-negative bacteria than from Gram-positive bacteria. MDR prevalence has been shown to be alarmingly high (55.7%). Only previous exposure to UTI and the past history of malnutrition have been found to have statistically significant relationship with the development of UTI in children. Therefore, precise isolate identification and careful antibiotic selection based on clinical data are crucial to mitigating the rapid evolution of drug-tolerant bacterial infections.

## Figures and Tables

**Table 1:** Sociodemographic characteristics of children attending Debre Tabor Comprehensive Specialized Hospital, Northwest Ethiopia.

Variables	Category	Frequency (*n*)	Percentage (%)
Gender	Male	111	50.5
	Female	109	49.5

Age (years)	<5	87	39.5
	5–10	88	40
	11–15	45	20.5

Residence	Urban	142	64.5
	Rural	78	35.5

Occupation (family)	Civil servant	82	37.3
	Farmer	85	38.6
	Merchant	38	17.3
	Other^#^	15	6.8

Education level (children)	No formal education	70	31.8
	Kindergarten	47	21.4
	Primary school	103	46.8

Education level (family)	Illiterate	25	11.4
	Primary school	76	34.5
	Secondary	66	30
	Tertiary	53	24.1

^#^Private worker, non-employed, housewife, and retired persons.

**Table 2:** Clinical characteristics of children who were clinically suspected of UTI in Debre Tabor Comprehensive Specialized Hospital, Northwest Ethiopia.

Explanatory variables	Category	Frequency (*n*)	Percentage (%)
Patient setting	Inpatient	66	30
	Outpatient	154	70

Previous history of catheterization	Yes	55	25
	No	165	75

Length of catheterizations (yes = 55)	<One week	35	63.6
	≥One week	20	36.4

History of antimicrobial exposure	Yes	113	51.4
	No	107	48.6

Past history of UTI	Yes	80	36.4
	No	140	63.6

Male circumcision (*n* = 111)	Yes	67	60.3
	No	44	39.6

Previous hospitalization	Yes	96	43.6
	No	124	56.4

Prolonged hospitalization	Yes	71	32.3
	No	149	67.7

Prolonged ICU stay	Yes	62	28.2
	No	158	71.8

Presence of malnutrition	Yes	57	25.9
	No	163	74.1

History of diabetes mellitus	Yes	73	33.2
	No	147	66.8

Presence of other chronic diseases	Yes	95	43.2
	No	125	56.8

UTI, urinary tract infection; ICU, intensive care unit.

**Table 3:** Bacterial profiles detected from the urine culture of pediatric patients visiting Debre Tabor Comprehensive Specialized Hospital, Northwest Ethiopia.

Bacterial types	Frequency (*n)*	Percentage (%)
Gram-positive	28	40
*S. aureus*	13	18.6
CoNS	9	12.9
*Enterococcus* spp.	6	8.5
Gram-negative	42	60
*E. coli*	19	27.1
*K. pneumoniae*	9	12.9
*Citrobacter* spp.	4	5.7
*Acinetobacter* spp.	3	4.3
*P. aeruginosa*	7	10
Total	70	100

CoNS: coagulase-negative staphylococci.

**Table 4:** Antimicrobial susceptibility pattern for the Gram-positive bacteria isolated from urine cultures of the pediatric patients at Debre Tabor Comprehensive Specialized Hospital.

Bacterial isolates	Antimicrobial agents tested (*n* = 10)										
	P	AMP no.(%)	PEN no. (%)	ERY no. (%)	SXT no. (%)	CIP no. (%)	TET no. (%)	CHL no. (%)	CN no. (%)	NIT no. (%)	CLN no. (%)
*S. aureus* (*n* = 13)	S	—	2 (15.4)	4 (30.8)	1(7.7)	6 (46.1)	3 (23.1)	4 (30.8)	7 (53.8)	0 (0)	13 (100)
	I	—	0 (0)	0 (0)	0 (0)	2 (15.4)	0 (0)	1 (7.7)	0 (0)	1 (7.7)	0 (0)
	R	—	11 (84.6)	9 (69.2)	12 (92.3)	5 (38.5)	10 (76.9)	8 (61.5)	6 (46.1)	12 (92.3)	0 (0)

CoNS (*n* = 9)	S	—	0 (0)	7 (77.8)	0 (0)	8 (88.9)	5 (55.6)	3 (33.3)	8 (88.9)	1 (11.1)	7 (77.8)
	I	—	0 (0)	1 (11.1)	0 (0)	0 (0)	0 (0)	4 (44.4)	0 (0)	0 (0)	0 (0)
	R	—	9 (100)	1 (11.1)	9 (100)	1 (11.1)	4 (44.4)	2 (22.2)	1 (11.1)	8 (88.9)	1 (11.1)

*Enterococcus* spp. (*n* = 6)	S	5 (83.3)	0 (0)	2 (33.3)	—	4 (66.7)	3 (50)	0 (0)	—	1 (16.7)	—
	I	0 (0)	0 (0)	0 (0)	—	1 (16.7)	0 (0)	0 (0)	—	0 (0)	—
	R	1 (16.7)	6 (100)	4 (66.7)	—	1 (16.7)	3 (50)	6 (100)	—	5 (83.3)	—

Total (%)	S	5 (17.9)	2 (7.1)	13 (46.4)	1 (3.6)	18 (64.3)	11 (39.3)	7 (25)	15 (53.6)	2 (7.1)	20 (71.4)
	I	0	0	1 (3.6)	0	3 (10.7)	0	5 (17.9)	0	1 (3.6)	0
	R	1 (3.6)	26 (92.9)	14 (50)	21 (75)	7 (25)	17 (60.7)	16 (57.1)	7 (25)	25 (89.3)	1 (3.6)

“—” denotes not applicable; P, patterns; CHL, chloramphenicol (30 *μ*g); CIP, ciprofloxacin (5 *μ*g); CN, gentamicin (10 *μ*g); NIT, nitrofurantoin (300 *μ*g); TET, tetracycline (30 *μ*g); SXT, trimethoprim-sulfamethoxazole (1.25/23.75 *μ*g); PEN, penicillin (10 units); CLN, clindamycin (2 *μ*g); AMP, ampicillin (10 *μ*g); ERY, erythromycin (15 *μ*g); S, sensitive; I, intermediate; R, resistant.

**Table 5:** Antimicrobial susceptibility pattern for the Gram-negative bacteria isolated from urine cultures of the pediatric patients visiting Debre Tabor Comprehensive Specialized Hospital.

Bacterial isolates	Antimicrobial agents tested (*n* = 11)											
	P	AMC no (%)	CIP no (%)	MER no (%)	AMP no (%)	CN no (%)	NIT no (%)	TET no (%)	CHL no (%)	SXT no (%)	CTR no (%)	CAZ no (%)
*E. Coli* (*n* = 19)	S	12 (63.2)	3 (15.8)	19 (100)	9 (47.4)	5 (26.3)	0 (0)	2 (10.5)	6 (31.6)	1 (5.3)	16 (84.2)	11 (57.9)
	I	0 (0)	0 (0)	0 (0)	0 (0)	2 (10.5)	0 (0)	1 (5.3)	0 (0)	0 (0)	1 (5.3)	0 (0)
	R	7 (36.8)	16 (84.2)	0 (0)	10 (52.6)	12 (63.2)	19 (100)	16 (84.2	13 (68.4)	18 (94.7)	2 (10.5)	8 (42.1)

*K. pneumoniae* (*n* = 9)	S	2 (22.2)	0 (0)	7 (77.8)	3 (33.3)	0 (0)	0 (0)	2 (22.2)	0 (0)	0 (0)	5 (55.6)	7 (77.8)
	I	0 (0)	0 (0)	0 (0)	2 (22.2)	0 (0)	0 (0)	0 (0)	4 (44.4)	0 (0)	2 (22.2)	0 (0)
	R	7 (77.8)	9 (100)	2 (22.2)	4 (44.4)	9 (100)	9 (100)	7 (77.8)	5 (55.6)	9 (100)	2 (22.2)	2 (22.2)

*Citrobacter* spp. (*n* = 4)	S	2 (50)	2 (50)	4 (100)	0 (0)	3 (75)	1 (25)	0 (0)	1 (25)	0 (0)	3 (75)	1 (25)
	I	1 (25)	0 (0)	0 (0)	0 (0)	1 (25)	1 (25)	0 (0)	0 (0)	0 (0)	0 (0)	1 (25)
	R	1 (25)	2 (50)	0 (0)	4 (100)	0 (0)	2 (50)	4 (100)	3 (75)	4 (100)	1 (25)	2 (50)

*Acinetobacter* spp. (*n* = 3)	S	—	0 (0)	3 (100)	—	0 (0)	—	—	—	0 (0)	1 (33.3)	0 (0)
	I	—	0 (0)	0 (0)	—	0 (0)	—	—	—	0 (0)	0 (0)	1 (33.3)
	R	—	3 (100)	0 (0)	—	3 (100)	—	—	—	3 (100)	2 (66.7)	2 (66.7)

*P. aeruginosa* (*n* = 7)	S	—	3 (42.9)	6 (85.7)	—	1 (14.3)	—	—	—	—	—	5 (71.4)
	I	—	2 (28.6)	0 (0)	—	2 (28.6)	—	—	—	—	—	0 (0)
	R	—	2 (28.6)	1 (14.3)	—	4 (57.1)	—	—	—	—	—	2 (28.6)

Total (%)	S	16 (38.1)	8 (19)	39 (92.9)	12 (28.6)	9 (21.4)	1 (2.4)	4 (9.5)	7 (16.7)	1 (2.4)	25 (59.5)	24 (57.1)
	I	1 (2.4)	2 (4.7)	0	2 (4.7)	5 (11.9)	1 (2.4)	1 (2.4)	4 (9.5)	0	3 (7.1)	2 (4.7)
	R	15 (35.7)	32 (76.2)	3 (7.1)	18 (42.9)	28 (66.7)	30 (71.4)	27 (64.3)	21 (50)	34 (81)	7 (16.7)	18 (42.9)

“—” denotes not applicable; P, patterns; AMC, amoxicillin-clavulanate acid (20/10 *μ*g); AMP, ampicillin (10 *μ*g); CTR, ceftriaxone (30 *μ*g); CHL, chloramphenicol (30 *μ*g); CIP, ciprofloxacin (5 *μ*g); CN, gentamicin (10 *μ*g); MER, meropenem (10 *μ*g); NIT, nitrofurantoin (300 *μ*g); TET, tetracycline (30 *μ*g); SXT, trimethoprim-sulfamethoxazole (1.25/23.75 *μ*g); CAZ, ceftazidime (30 *μ*g); S, sensitive; I, intermediate; R, resistant.

**Table 6:** Multidrug resistance pattern of bacterial uropathogens recovered from the urine culture of children attending Debre Tabor Comprehensive Specialized Hospital, Northwest Ethiopia.

Bacterial types	Total isolates no (%)	*R*_0_ no (%)	*R*_1_ no (%)	*R*_2_ no (%)	*R*_3_ no (%)	*R*_4_ no (%)	≥*R*_5_ no (%)
Gram-positive	28 (40)	—	2 (7.1)	6 (21.4)	10 (35.7)	6 (21.4)	4 (14.3)
*S. aureus*	13 (46.4)	—	1 (7.7)	4 (30.8)	3 (23.1)	5 (38.5)	1 (7.7)
CoNS	9 (32.1)	—	—	2 (22.2)	4 (44.4)	—	1 (11.1)
*Enterococcus* spp.	6 (21.4)	—	1 (16.7)	—	3 (50)	1 (16.7)	2 (33.3)
Gram-negative	42 (60)	1 (2.4)	3 (7.1)	19 (45.2)	13 (31)	4 (9.5)	2 (4.8)
*E. coli*	19 (45.2)	—	2 (10.5)	7 (36.8)	2 (10.5)	1(5.3)	1(5.3)
*K. pneumoniae*	9 (21.4)	—	1 (11.1)	5 (55.6)	4 (44.4)	1 (11.1)	—
*Citrobacter* spp.	4 (9.5)	1 (25)	1 (25)	2 (50)	3 (75)	—	—
*Acinetobacter* spp.	3 (7.1)	—	—	2 (28.6)	3(100)	2 (66.7)	—
*P. aeruginosa*	7 (16.7)	—	—	3 (42.9)	1 (14.3)	—	1 (14.3)
Total	70 (100)	1 (1.4)	5 (7.1)	25 (35.7)	23 (32.9)	10 (14.3)	6 (8.6)

*R*_0_, no antibiotic resistance; *R*_1_, resistance to one; *R*_2_, resistance to two; *R*_3_, resistance to three; *R*_4_, resistance to four; ≥*R*_5_, resistance to five and more than five antimicrobials belonging to a different class.

**Table 7:** Bivariate and multivariate logistic regression analysis of the factors associated with UTI among children attending Debre Tabor Comprehensive Specialized Hospital, Northwest Ethiopia.

Variables	Category	Frequency (%)	UTI status		Logistic regression analysis	
					Bivariate COR (95% CI)	Multivariate AOR (95% CI)
			Positive no (%)	Negative no (%)		
Gender	Male	111 (50.5)	31 (27.9)	80 (72.1)	1	1
	Female	109 (49.5)	39 (35.8)	70 (64.2)	1.01 (0.02–1.72)	0.67 (0.02–1.86)

Age (years)	<5	8 7 (39.5)	34 (39.1)	53 (60.9)	1.78 (0.55–5.58)	1.41 (0.86–5.97)
	5–10	88 (40)	19 (21.6)	69 (78.4)	1.35 (0.44–4.65)	1.21 (0.33–4.32)
	11–15	45 (20.5)	17 (37.8)	28 (62.2)	1	

Residence	Urban	142 (64.5)	26 (18)	116 (82)	1	1
	Rural	78 (35.5)	44 (56.4)	34 (43.6)	1.07 (0.08–1.12)	0.88 (0.32–2.11)

Occupation (family)	Civil servant	82 (37.3)	18 (22)	64 (78)	1	1
	Farmer	85 (38.6)	22 (26)	63 (74)	2.09 (1.66–2.22)	0.86 (0.03–1.77)
	Merchant	38 (17.3)	15 (39.5)	23 (60.5)	0.19 (0.06–1.32)	1.06 (0.03–2.07)
	Other^**#**^	15 (6.8)	15 (100)	0	1.66 (0.24–1.98)	0.92 (0.05–1.98)

Education level (children)	No formal education	70 (31.8)	23 (32.9)	47 (67.1)	1.11 (0.02–1.86	0.44 (0.03–1.26)
	Kindergarten	47 (21.4)	13 (27.7)	34 (72.3)	0.04 (0.01–0.83)	0.21 (0.01–0.89)
	Primary school	103 (46.8)	34 (33)	69 (67)	1	1

Education level (family)	Illiterate	25 (11.4)	8 (32)	17 (68)	0.13 (0.02–0.88)	1.01 (0.06–1.01)
	Primary school	76 (34.5)	26 (34.2)	50 (65.8)	0.25 (0.11–0.71)	1.23 (0.99–2.76)
	Secondary	66 (30)	19 (28.8)	47 (71.2)	1.55 (0.82–2.44)	0.85 (0.02–1.19)
	Tertiary	53 (24.1)	17 (32.1)	36 (67.9)	1	

History of catheterization	Yes	55 (25)	47 (85.5)	8 (14.5)	0.45 (0.21–1.38)	3.22 (1.06–7.31)
	No	165 (75)	23 (13.9)	142 (86.1)	1	1

Previous history of UTI	Yes	80 (36.4)	52 (65)	28 ((35)	0.27 (0.09–2.44)	1.04 (0.39–−2.75)^*∗*^
	No	140 (63.6)	18 (12.9)	122 (87.1)	1	

History of antimicrobial treatment	Yes	113 (51.4)	27 (23.9)	86 (76.1)	0.52 (0.143–0.96)	0.96 (0.31–2.58)
	No	107 (48.6)	43 (40.2)	64 (59.8)	1	

Presence of malnutrition	Yes	57 (25.9)	42 (73.7)	15 (26.3)	0.46 (0.16–1.48)	2.18 (0.14–−5.135)^*∗*^
	No	163 (74.1)	28 (17.2)	135 (82.8)	1	1

Patient setting	Inpatient	66 (30)	31 (47)	35 (53)	2.11 (1.02–4.58)	0.12 (0.08–2.37)
	Outpatient	154 (70)	39 (25.3)	115 (74.7)	1	

Prolonged ICU stays	Yes	62 (28.2)	55 (88.7)	7 (11.3)	1.37 (0.14–3.92)	1.45 (0.29, 4.48)
	No	158 (71.8)	15 (9.5)	143 (90.5)	1	1

Length of catheterizations (yes = 55)	<One week	35 (63.6)	17 (48.6)	18 (51.4)	1	
	≥One week	20 (36.4)	9 (45)	11 (55)	2.09 (1.04–3.09)	2.01 (0.99–2.66)

Male circumcision (*n* = 111)	Yes	67 (60.3)	37 (55.2)	30 (44.8)	0.33 (0.13–1.45)	1.18 (1.01–1.73)
	No	44 (39.6)	23 (52.3)	21 (47.7)	1	

Previous hospitalization	Yes	96 (43.6)	55 (57.3)	41 (42.7)	0.22 (0.06–1.17)	1.08 (0.09–1.88)
	No	124 (56.4)	15 (12.1)	109 (87.9)	1	1

Prolonged hospitalization	Yes	71 (32.3)	26 (36.6)	45 (63.4)	1.02 (0.11–2.05)	0.99 (0.31–1.86)
	No	149 (67.7)	44 (29.5)	105 (70.5)	1	1

History of diabetic matatus	Yes	73 (33.2)	19 (26)	54 (74)	1.43 (1.26–2.01)	0.83 (0.77–1.78)
	No	147 (66.8)	51 (34.7)	96 (65.3)	1	

Presence of other chronic diseases	Yes	95 (43.2)	30 (31.6)	65 (68.4)	0.55 (0.07–1.12)	0.78 (0.17–2.09)
	No	125 (56.8)	40 (32)	85 (68)	1	1

COR, crude odds ratio; AOR, adjusted odds ratio; ^∗^denotes significance at a *p* value <0.05. ^#^Private worker, non-employed, housewife, and retired persons.

## Data Availability

The data used to support the findings of this study are included within the article.

## Abbreviations

AMR: Antimicrobial resistance
AST: Antimicrobial susceptibility testing
CLED: Cystine lactose electrolyte-deficient
CLSI: Clinical and Laboratory Standards Institute
DTCSH: Debre Tabor Comprehensive Specialized Hospital
MDR: Multidrug resistance
MHA: Mueller–Hinton agar
UTI: Urinary tract infection.
